# Real-world effectiveness and safety of sofosbuvir and nonstructural protein 5A inhibitors for chronic hepatitis C genotype 1, 2, 3, 4, or 6: a multicentre cohort study

**DOI:** 10.1186/s12876-020-01196-0

**Published:** 2020-03-05

**Authors:** Phunchai Charatcharoenwitthaya, Virasak Wongpaitoon, Piyawat Komolmit, Wattana Sukeepaisarnjaroen, Pisit Tangkijvanich, Teerha Piratvisuth, Theeranun Sanpajit, Chinnavat Sutthivana, Chalermrat Bunchorntavakul, Abhasnee Sobhonslidsuk, Soonthorn Chonprasertsuk, Chotipong Siripipattanamongkol, Supatsri Sethasine, Tawesak Tanwandee, The THASL Collaborating Group for the Study of the Use of Direct-acting Antivirals for Chronic Hepatitis C

**Affiliations:** 1 Faculty of Medicine, Siriraj Hospital, Mahidol University, Bangkok, Thailand; 2 Bumrungrad International Hospital, Bangkok, Thailand; 3 Faculty of Medicine, Chulalongkorn University, Bangkok, Thailand; 4 Faculty of Medicine, Khon Kaen University, Khon Kaen, Thailand; 5 Faculty of Medicine, Prince of Songkla University, Songkhla, Thailand; 6 Phramongkutklao Hospital, Bangkok, Thailand; 7 Bhumibol Adulyadej Hospital, Bangkok, Thailand; 8 Rajavithi Hospital, Bangkok, Thailand; 9 Faculty of Medicine, Ramathibodi Hospital, Mahidol University, Bangkok, Thailand; 10 Faculty of Medicine, Thammasat University, Pathumthani, Thailand; 11 Chiangrai Prachanukroh Hospital, Chiangrai, Thailand; 12 BMA Medical College and Vajira Hospital, Bangkok, Thailand

**Keywords:** Hepatitis C, Sofosbuvir, Daclatasvir, Velpatasvir, Effectiveness, Safety

## Abstract

**Background:**

We investigated real-world effectiveness and safety of sofosbuvir and the nonstructural protein 5A inhibitors in the treatment of patients infected with hepatitis C virus (HCV) genotypes 1, 2, 3, 4, or 6.

**Methods:**

We analyzed data from 1021 patients with HCV infection (506 with genotype 1; 16 with genotype 2; 314 with genotype 3; 13 with genotype 4; 166 with genotype 6) who received 12 to 24 weeks of daclatasvir plus sofosbuvir (*n* = 767), ledipasvir/sofosbuvir (*n* = 197), or sofosbuvir/velpatasvir (*n* = 57), with or without ribavirin in 12 centers across Thailand to estimate sustained virologic response at post-treatment week 12 (SVR12).

**Results:**

Overall, SVR12 rate was 98.0% (95% confidence interval [CI], 96.7–98.8%) with daclatasvir plus sofosbuvir, 97.9% (95% CI, 94.8–99.2%) with ledipasvir/sofosbuvir, and 96.5% (95% CI, 88.1–99.0%) with sofosbuvir/velpatasvir. SVR12 was achieved by 99.2% (95% CI, 97.9–99.7%) of subjects with genotype 1 infection, 100% (95% CI, 78.5–100%) of those with genotype 2 infection, 96.7% (95% CI, 94.0–98.2%) of those with genotype 3 infection, 90.9% (95% CI, 62.3–98.4%) of those with genotype 4 infection, and 96.7% (95% CI 92.5–98.6%) of those with genotype 6 infection. Patients with advanced liver disease were at risk of treatment failure. Only four patients discontinued treatment before week 4 due to non-hepatic adverse events.

**Conclusions:**

In this large cohort of patients with various HCV genotypes managed in the real-world practice setting, daclatasvir plus sofosbuvir, ledipasvir/sofosbuvir, and sofosbuvir/velpatasvir achieved high SVR rates with good safety profile, comparable to those observed in clinical trials.

## Background

Chronic hepatitis C virus (HCV) infection is recognized as a common cause of chronic liver disease leading to cirrhosis, hepatocellular carcinoma (HCC), and liver-related mortality across the world [1]. A sustained virologic response (SVR) after effective antiviral treatment is associated with a decreased risk in liver disease progression and its complications, including portal hypertension, decompensation, HCC, and death [2, 3]. The arrival of direct-acting antiviral agents (DAAs) has drastically changed HCV treatment by increasing the likelihood of SVR and shortening the duration of treatment [4]. Several sofosbuvir (SOF)-based regimens have demonstrated their excellent efficacy and safety for treating chronic HCV infection. In particular, combining SOF with the nonstructural protein 5A (NS5A) inhibitors, e.g., ledipasvir (LDV), daclatasvir (DAC), or velpatasvir (VEL) has shown remarkable efficacy with SVR rates at post-treatment week 12 exceeding 95% in clinical trials [5–13]. Even though the demonstrated safety and efficacy of the combination therapy in registrational trials is paramount, the continued safety and effectiveness of these regimens in diverse clinical care settings are of great importance.

While the real-world data of DAA therapy for chronic hepatitis C have been reported mostly from western countries [14–22], there is a paucity of Asian data concerning DAAs [23–27]. Management of HCV in Asian countries continues to be challenging for a variety of reasons. The availability and approvals for DAAs in Asia lag behind those in Europe and North America. HCV genotype in this geographic region is disparate [28]. Accordingly, there is an unmet need to understand the management of HCV with all-oral DAAs in Asian countries, including Thailand. Such data on effectiveness and safety in clinical practice are essential for guiding patients and physicians in making decisions about treatment regimens, as well as informing health care policy around treatment coverage.

We reported here on our multicenter experience, which included a large number of patients infected with HCV genotypes 1, 2, 3, 4, or 6, who received SOF and NS5A inhibitors combination therapy, with or without ribavirin. The primary aim of this study was to determine the effectiveness and safety of SOF-based treatment regimens among clinically relevant patient subgroups.

## Methods

### Study population

This was a retrospective, non-interventional, national, multicenter study evaluating antiviral treatment of HCV-infected patients in routine clinical practice. The study obtained data from all adult patients chronically infected with HCV genotypes 1, 2, 3, 4, or 6 undergoing treatment with SOF and the NS5A inhibitors ± ribavirin (RBV) in 12 Thai centers between December 2015 and August 2017. Chronic HCV infection was defined as the presence of HCV antibody for more than six months and detectable serum HCV RNA.

### Treatment regimens

The following treatment regimens were used according to the national guideline and availability of DAAs: 1) DAC + SOF ± RBV for 12–24 weeks, 2) LDV/SOF ± RBV for 12–24 weeks or 3) SOF/VEL ± RBV for 12 weeks. The choice of antiviral therapy was entirely at the discretion of the treating physician concerning HCV genotypes, the patient’s prior treatment experience, and the presence of cirrhosis. For those receiving RBV, dosing varied across patients and treatment centers; however, for most patients, RBV was administered according to body weight (< 65 kg, 800 mg daily; 65 to 85 kg, 1000 mg daily; > 85 kg, 1200 mg daily in 2 divided doses). In patients with decompensated cirrhosis, the daily dose of RBV was started with 600 mg and can be titrated up according to patients’ tolerability.

### Measurements

Clinical and laboratory data were collected at baseline, and as available throughout the treatment period and the post-treatment follow-up. Laboratory data were collected per standard practice at the local laboratories. HCV RNA levels were measured using the COBAS AmpliPrep/COBAS TaqMan, version 2 (lower limit of detection, 15 IU/mL) (Roche, Branchburg, NJ) or the RealTime HCV assay (lower limit of detection, 12 IU/mL) (Abbott Molecular, Wiesbaden, Germany). HCV genotype and subtype was determined using the VERSANT HCV Genotype 2.0 assay (LiPA) (Siemens, Erlangen, Germany). The CKD-EPI equation calculated the estimated glomerular filtration rate (eGFR) [29], and chronic kidney disease was staged according to KDIGO guidelines [30].

The presence of cirrhosis before treatment was determined by liver biopsy examination showing cirrhosis (METAVIR stage 4), or a liver stiffness measurement of more than 12.5 kPa (transient elastography). Child-Turcotte-Pugh (CTP) score and the model of the end-stage liver disease (MELD) score were calculated at baseline and week 12 after treatment. Decompensated cirrhosis was defined as evidence of a prior or current diagnosis of ascites, spontaneous bacterial peritonitis, variceal hemorrhage, or hepatic encephalopathy, or concomitant medications with a specific use listed for these conditions. Adverse events, defined as any new symptom occurring during the HCV treatment period, were collected.

### Assessments

The primary efficacy endpoint was SVR, defined as undetectable HCV RNA 12 weeks after the end of treatment (SVR12). Virologic relapse was defined as a detectable HCV RNA level after the end of treatment in a patient who had had undetectable HCV RNA during treatment. Treatment failure was defined as detectable HCV RNA at any time during treatment or post-treatment follow-up. Patients who did not attend their scheduled follow-up visit 12 (or 24) weeks after the end of therapy were regarded as lost-to-follow-up.

### Statistical analysis

Demographic, disease characteristics, and laboratory values were analyzed by the HCV genotype for the evaluable population. The unadjusted rate of SVR12 was calculated for the modified intention-to-treat (mITT) population that included patients who received ≥1 dose of a DAA regimen but did not include those without virological failure who were lost to follow-up or who died from disorders unrelated to the treatment. Additional effectiveness for subgroups of interest, particularly based on treatment history, the presence of cirrhosis and decompensation, and HCV subgenotype, were analyzed. Two-sided 95% confidence intervals (CIs) of the SVR12 rates were calculated using exact binomial methods. Logistic regression analysis was used to identify any independent baseline factors influencing treatment failure. All statistical testing was done at the two-tailed α level of 0.05. The SPSS software package version 18.0 (SPSS Inc., Chicago, IL) was used for analysis.

## Results

### Patient characteristics

**Fig. 1:**
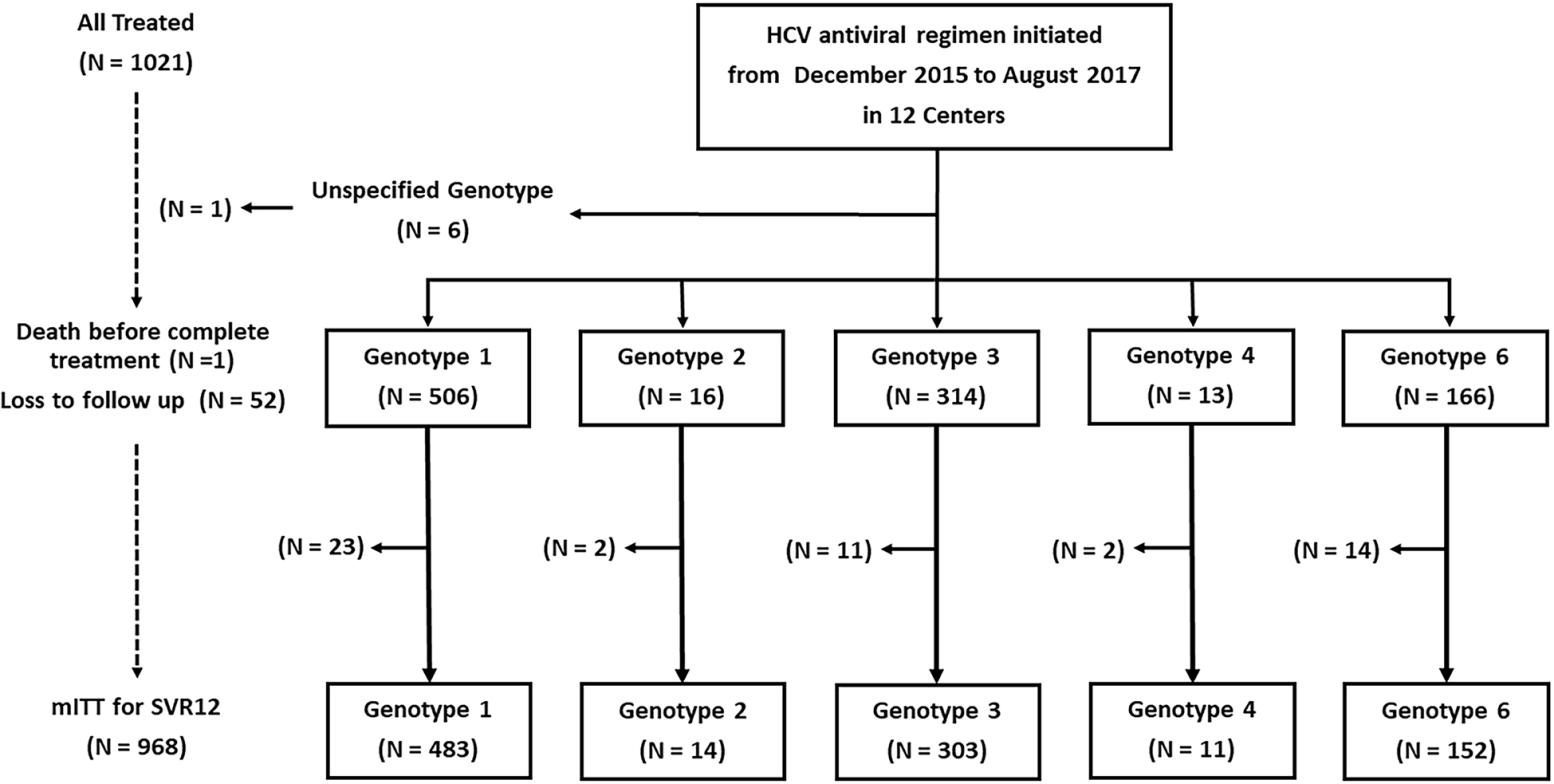
Derivation of the study population

The mean age of patients who initiated antiviral treatment was 57.3 ± 11.2 years, 45.2% were male, 5.3% were taking the proton pump inhibitors at baseline, and 46.2% were peginterferon plus RBV treatment-experienced including 2% experienced treatment failure with peginterferon and RBV plus boceprevir (Table 1). Patients were infected primarily with HCV genotypes 1 (49.5%), 3 (31%), or 6 (16%), as shown in Fig. 1, and 44.3% had HCV RNA ≥2 × 10^6^ IU/mL at baseline. Ninety-one patients underwent liver biopsy, and 44 (48.4%) of them had METAVIR stage 4. Five hundred and twenty-two patients (51.1%) had a diagnosis of cirrhosis, including 85 patients with CTP score ≥ 7 and 3 with MELD > 20. Low platelet counts < 100 × 10^9^/L, serum albumin < 3.5 g/dL, and stage 3–4 chronic kidney disease (eGFR < 60 mL/min/1.73 m^2^), were present in 18.9, 17.8 and 8.5% of the cohort, respectively.

**Table 1:** Baseline Characteristics of Patients Who Initiated Antiviral Therapy

Characteristics	All Genotypes	Genotype 1	Genotype 2	Genotype 3	Genotype 4	Genotype 6
All patients	1021	506	16	314	13	166
Age, year	57.3 ± 11.2	57.1 ± 11.2	55.8 ± 11.5	56.5 ± 10.8	55.7 ± 13.6	59.2 ± 11.4
Male, *n* (%)	461 (45.2%)	236 (46.6%)	8 (50.0%)	133 (42.4%)	4 (30.8%)	77 (46.4%)
Race/Ethnicity, *n* (%)						
Thai	774 (75.8%)	402 (79.5%)	0	282 (90%)	3 (23%)	84 (50.6%)
Asian	162 (15.9%)	71 (14%)	6 (37.5%)	24 (7.5%)	0	58 (34.9%)
Others	85 (8.3%)	33 (6.5%)	10 (62.5%)	8 (2.5%)	10 (77%)	24 (14.5%)
Treatment experienced, *n* (%)	472 (46.2%)	263 (52%)	2 (12.5%)	148 (47.1%)	7 (53.8%)	50 (30.1%)
HBV co-infection, *n* (%)	27 (2.6%)	12 (2.4%)	1 (6.3%)	14 (4.5%)	0	0
HIV co-infection, *n* (%)	21 (2.1%)	13 (2.6%)	0	8 (2.5%)	0	0
Liver transplant recipient, *n* (%)	31 (3.0%)	16 (3.2%)	1 (6.3%)	9 (2.9%)	1 (7.7%)	4 (2.4%)
Hepatocellular carcinoma, *n* (%)	28 (2.7%)	10 (2%)	1 (6.3%)	13 (4.1%)	1 (7.7%)	3 (1.8%)
Cirrhosis, *n* (%)	521 (51.0%)	244 (48.2%)	4 (25%)	203 (64.6%)	7 (53.8%)	63 (38%)
Child-Turcotte-Pugh class, *n* (%)						
A	436 (83.7%)	219 (90%)	2 (50%)	157 (77%)	4 (57%)	54 (86%)
B	78 (15%)	25 (10%)	2 (50%)	42 (21%)	3 (43%)	6 (9%)
C	7 (1.3%)	0	0	4 (2%)	0	3 (5%)
MELD score	8.1 ± 2.6	7.8 ± 2.2	7.2 ± 1.1	8.7 ± 3.0	8.5 ± 2.5	8.0 ± 3.2
Platelet count < 100 × 10^9^/μL, *n* (%)	193 (18.9%)	79 (15.6%)	0	84 (26.8%)	2 (15%)	28 (16.9%)
Albumin < 3.5 g/dL, *n* (%)	182 (17.8%)	69 (13.6%)	4 (25%)	76 (24.2%)	5 (38.5%)	27 (16.3%)
Total bilirubin > 1.1 mg/dL, *n* (%)	189 (18.5%)	87 (17.2%)	1 (16.7%)	77 (24.5%)	3 (23.1%)	21 (12.7%)
HCV RNA, mean ×10^6^ IU/mL	3.6 ± 5.9	3.5 ± 6.2	4.2 ± 6.0	3.0 ± 4.2	2.4 ± 3.1	5.2 ± 7.3
≥2 × 10^6^ IU/mL, *n* (%)	452 (44.3%)	228 (45.1%)	7 (43.8%)	118 (37.6%)	4 (30.8%)	93 (56.0%)
Creatinine clearance, mL/min/1.73 m^2^						
≥ 90	424 (41.5%)	210 (41.5%)	9 (56.3%)	126 (40.1%)	7 (53.8%)	69 (41.6%)
60–89	352 (34.5%)	178 (35.2%)	4 (25.0%)	111 (35.4%)	2 (15.4%)	56 (33.7%)
30–59	81 (7.9%)	40 (7.9%)	1 (6.2%)	25 (8.0%)	3 (23.1%)	12 (7.2%)
< 30	6 (0.6%)	3 (0.6%)	0	1 (0.3%)	0	2 (1.2%)
Not reported	158 (15.5%)	75 (14.8%)	2 (12.5)	51 (16.2%)	1 (7.7%)	27 (16.3%)

Twenty-eight patients (2.7%) had a treated HCC, which was classified as Barcelona Clinic Liver Cancer stage 0/A in 71% and stage B in 29%. They had a complete response to surgical resection and/or locoregional therapies at least six months before DAA therapy. Twenty-seven patients (2.6%) were coinfected with the hepatitis B virus (HBV), and only 14 of them had received nucleos(t) ide analogue therapy. All but two HBV/HCV coinfected individuals had undetectable HBV DNA at the start of DAA therapy. Twenty-one patients (2.1%) coinfected with human immunodeficiency virus (HIV) received antiretroviral therapy, mostly triple-drug regimens with two nucleoside analogues (*n* = 15) followed by protease inhibitor-based regimens (*n* = 4) and a non-nucleoside reverse transcriptase inhibitor-based regimen (*n* = 2). All patients had HIV RNA values of < 50 copies/mL and a CD4 cell count of ≥100 cells/mL without the need for dose adjustment of all antiretroviral regimens during HCV treatment. Thirty-one (3%) patients were liver transplant recipients; of them, 55% were cirrhotic (9.7% CTP class B 3.2% CTP class C), and one patient had fibrosing cholestatic hepatitis. The commonly used immunosuppressants in liver transplant recipients were tacrolimus (77%), mycophenolic acid (48%), and rapamycin inhibitors (32%).

### Treatment regimens

**Fig. 2:**
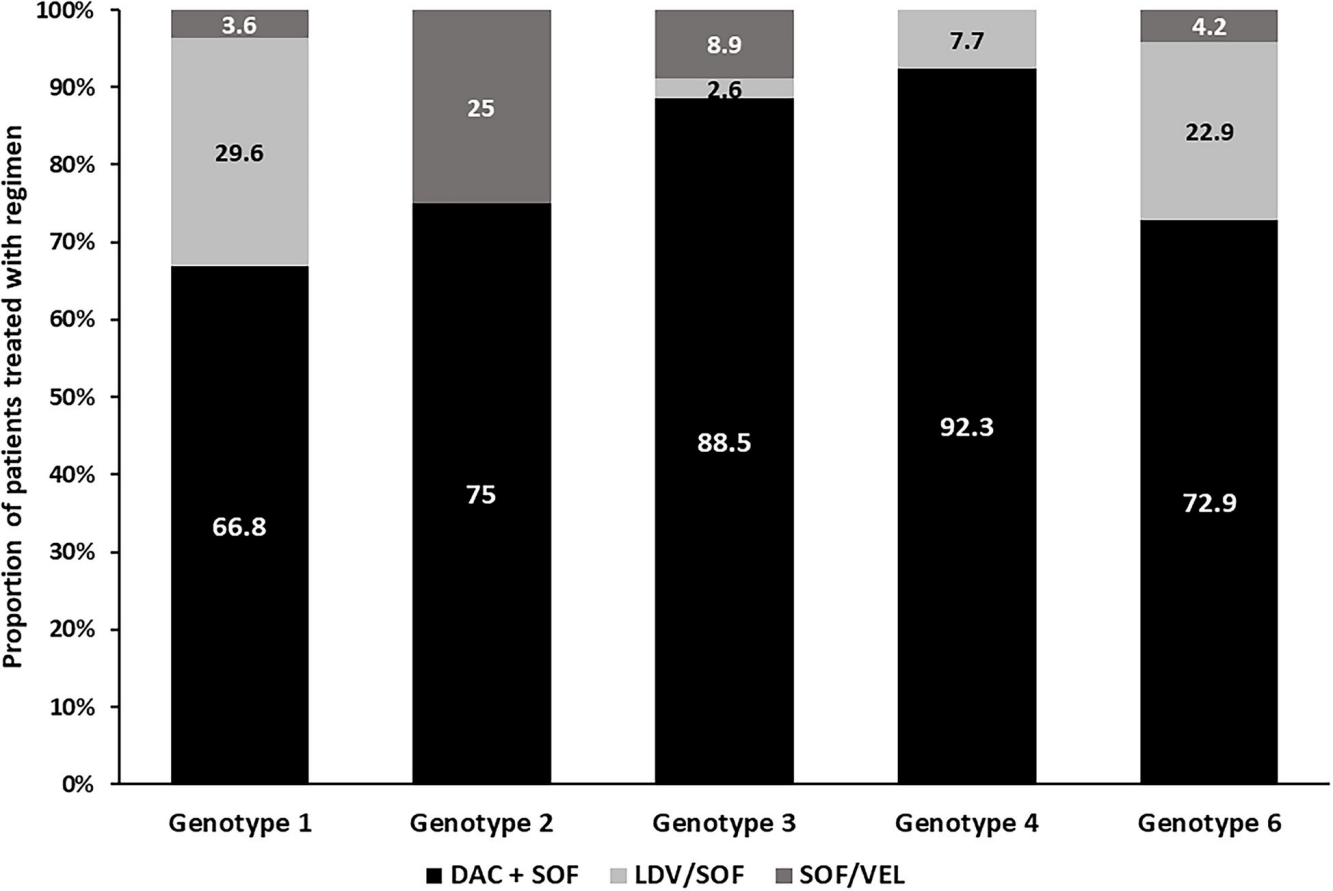
Distribution of HCV antiviral regimens by genotype

In Thailand, the first DAA regimen approved was a combination of DAC and SOF. Other fixed-dose combinations such as LED/SOF and SOF/VEL were later available for different HCV genotypes. Hence, the majority of HCV genotype 1-infected patients (*n* = 506) were primarily treated with DAC + SOF (27.7%) or in combination with RBV (39.1%), followed by LDV/SOF with (15.2%) or without (14.4%) RBV, while SOF/VEL alone (2.4%) or with RBV (1.2%) were used much less frequently (Fig. 2). Genotype 2–infected patients (*n* = 16) were treated with DAC + SOF (50%) or in combination with RBV (25%), and SOF/VEL monotherapy (25%). The majority of genotype 3–infected patients (*n* = 314) were treated with DAC + SOF with (60.8%) or without RBV (27.7%), followed by SOF/VEL with (2.9%) or without RBV (6%), and LDV/SOF with RBV (2.6%). Genotype 4–infected patients (*n* = 13) were treated mostly with DAC + SOF alone (46.1%) or in combination with RBV (46.1%), while the only patient (7.7%) received LDV/SOF with RBV. The majority of genotype 6-infected patients (*n* = 166) were treated with DAC + SOF with (31.3%) or without (41.6%) RBV, followed by LDV/SOF alone (12%) or in combination with RBV (10.9%), and the remaining patients were treated with SOF/VEL alone (3%) or in combination with RBV (1.2%). Patients infected with unspecified genotypes (*n* = 6) were all treated with DAC + SOF with (67%) or without (33%) RBV.

The majority of cirrhotic patients were treated with 12 weeks of DAC + SOF with (43.3%) or without RBV (10.7%), followed by 12 weeks of LDV/SOF with (14.2%) or without RBV (2.5%), and 12 weeks of SOF/VEL with (3.1%) or without RBV (4.2%). Extending treatment duration were used in 115 cirrhotic patients with 16 weeks of DAC + SOF with RBV (5.8%), 24 weeks of DAC + SOF with (9.4%) or without RBV (4.4%), and 24 weeks of LDV/SOF with (0.6%) or without RBV (1.9%). Liver transplant recipients were treated mostly with 12 weeks of DAC + SOF with (77.4%) or without RBV (16.3%), and two patients (6.4%) received 24 weeks of LDV/SOF with RBV. All six patients with eGFR < 30 mL/min/1.73 m^2^ at baseline started antiviral therapy with a daily dose of SOF 400 mg, and eGFR remained stable without dose adjustment.

### Treatment outcomes

Overall, SVR12 was achieved by 97.9% (95% CI, 96.8%–98.7%) of the 968 patients in the mITT analysis, including 98.0% (95% CI, 96.7%–98.8%) of patients treated with DCV + SOF, 97.9% (95% CI, 94.8%–99.2%) of patients treated with LDV/SOF, and 96.5% (95% CI, 88.1%–99.0%) of those treated with SOF/VEL. The SVR12 rates were generally comparable across treatment regimens. The SVR12 rates were achieved by 99.2% of patients (479 of 483) infected with HCV genotype 1, all 14 patients (100%) with genotype 2, 96.7 of patients (293 of 303) with genotype 3, 90.9% of patients (10 of 11) with genotype 4, 96.7% of patients (147 of 152) infected with genotype 6, and all five patients (100%) with unspecified genotype. Besides, SVR12 was achieved by 90.5% of HIV/HCV coinfected patients (19 of 21), and all 25 HBV/HCV coinfected patients (100%) without evidence of HBV reactivation. SVR12 was achieved by 96.3% of 27 liver transplant recipients without an episode of acute rejection. The SVR12 rates were 92.3% for 26 patients with HCC, and 97.5% for 479 cirrhotic patients without HCC (*p* = 0.16). Renal insufficiency had no impact on virologic response; SVR12 rates were achieved by 98.5% of 342 patients with eGFR 60–89 mL/min/1.73 m^2^, 98.7% of 78 patients with eGFR 30–59 mL/min/1.73 m^2^ and 100% of 6 patients with eGFR < 30 mL/min/1.73 m^2^.

Rates of virologic response were high regardless of cirrhosis status or liver disease severity, as indicated by low platelet counts or albumin levels. SVR12 was achieved by 97.2% (490 of 504) of patients with cirrhosis (95.8–98.0% with DCV + SOF ± RBV; 95.7–97.3% with LDV/SOF ± RBV; 90.9–100% with SOF/VEL ± RBV), 94.6% (156 of 165) of cirrhosis patients with platelet counts < 100 × 10^9^/L, 94.5% (137 of 145) of those with albumin < 3.5 g/dL and 92.8% (129 of 139) of those with total bilirubin ≥1.2 mg/dL. The SVR12 rate was significantly lower in patients with decompensated cirrhosis (CTP class B/C) compared with those with CTP class A (91.0% vs. 98.5%; *p* < 0.001). In decompensated cirrhosis patients, improvements in MELD scores were observed in 30 (65%) of 46 patients with SVR12 who had measurements for MELD scores at both baseline and week 12 of follow-up. Of the patients with SVR12 and MELD improvements, 24.4% of them yet remained decompensated post-treatment.

**Fig. 3:**
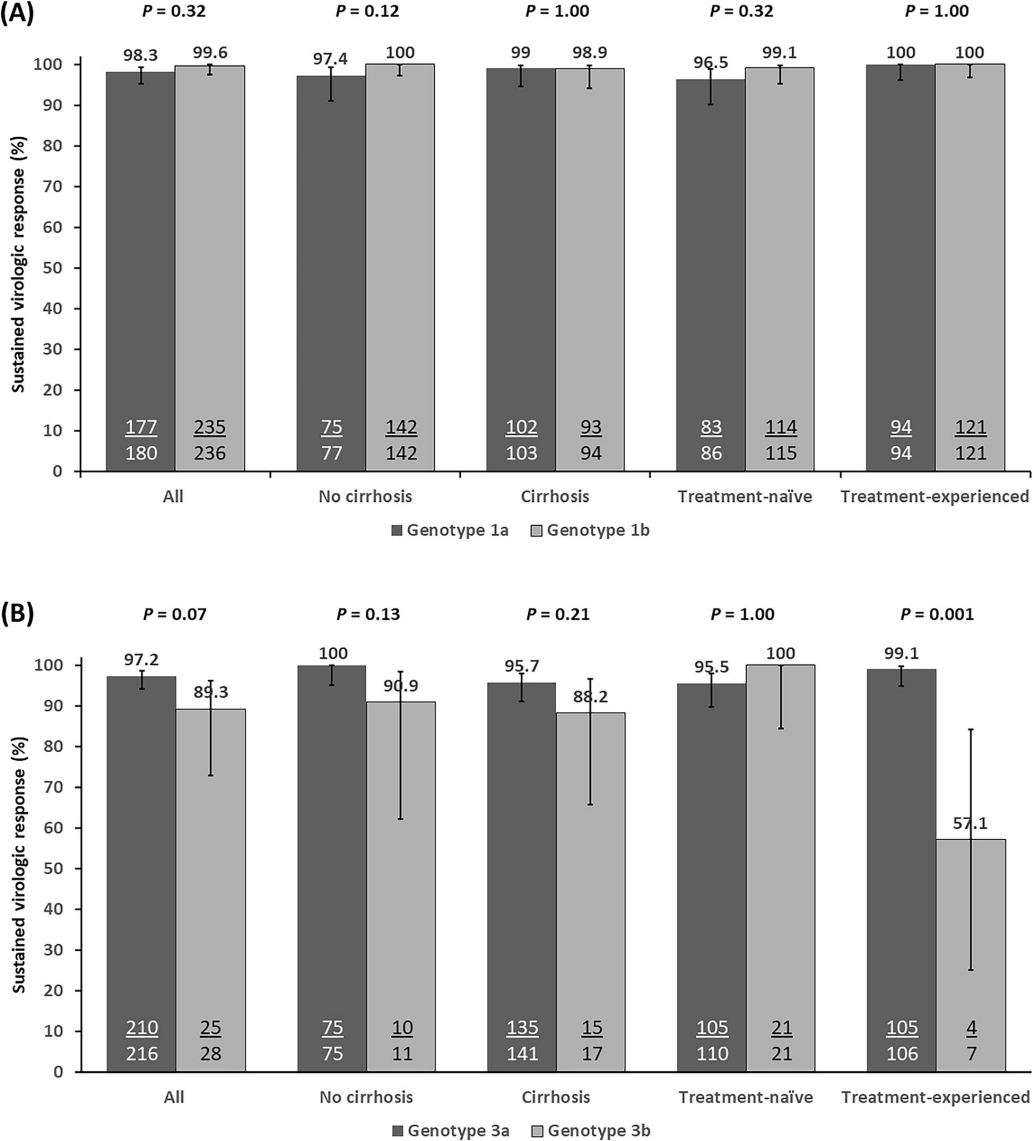
Sustained virologic response in patients with HCV genotype 1 and 3 infections. SVR12 (mITT analysis) rates by HCV subgenotype are shown according to baseline cirrhosis status and prior HCV therapy in patients with HCV genotype 1 **a** and 3 **b** infection patients. Error bars indicate 95% confidence intervals

Among genotype 1–infected patients, all SOF-based combinations had similarly high SVR12 rates, ranging from 98.7 to 100%, with no significant difference between DAC + SOF, LDV/SOF, and SOF/VEL as shown in Table 2. All genotype 1–infected patients with cirrhosis and prior treatment experience achieved SVR12 with 12 weeks of SOF-based regimens with or without RBV. Similarly, SVR12 was achieved in all treatment-experienced patients with cirrhosis who received extending treatment duration to 16 or 24 weeks. In patients with available subtype data (*n* = 416), comparable SVR12 rates were observed in patients with genotype 1a (98.3%, 177 of 180) and genotype 1b (99.6%, 235 of 236) patients. Rates of SVR12 between both subtypes were similar when data were analyzed by the presence or absence of cirrhosis and prior treatment experience (Fig. 3a). Although genotype 1b patients had slightly higher SVR than genotype 1a patients treated with LDV/SOF regimen (100% vs. 96.2%), there was no difference in SVR12 rates of genotype 1a and 1b for DAC + SOF and SOF/VEL regimens.

**Table 2:** Sustained Virologic Response Rates (95% Confidence Intervals) in Patients infected with HCV Genotype 1

	Genotype 1 SVR, % (95% CI)	Genotype 1a SVR, % (95% CI)				Genotype 1b SVR, % (95% CI)			
	All patients	All patients	LDV/SOF	DAC + SOF	SOF/VEL	All patients	LDV/SOF	DAC + SOF	SOF/VEL
Patients who complete, N	483	180	52	117	11	236	86	143	7
All durations	99.2 (97.9–99.7)	98.3 (95.2–99.4)	96.2 (87.0–98.9)	99.1 (95.3–99.8)	100 (74.1–100)	99.6 (97.6–99.9)	100 (95.7–100)	99.3 (96.1–99.9)	100 (64.6–100)
12-wk treatment	99.1 (97.7–99.7)	98.2 (94.7–99.4)	95.8 (86.0–98.8)	99.0 (94.8–99.8)	100 (74.1–100)	99.6 (97.5–99.9)	100 (95.5–100)	99.3 (96.0–99.9)	100 (64.6–100)
16-wk treatment	100 (67.6–100)	100 (34.2–100)	NA	100 (34.2–100)	NA	100 (51.0–100)	NA	100 (51.0–100)	NA
24-wk treatment	100 (87.1–100)	100 (79.6–100)	100 (51.0–100)	100 (74.1–100)	NA	100 (64.6–100)	100 (56.6–100)	100 (34.2–100)	NA
SVR in the following subgroups									
No cirrhosis	99.2 (97.1–99.8)	97.4 (91.0–99.3)	96.0 (80.5–99.3)	98.0 (89.5–99.6)	100 (34.2–100)	100 (97.4–100)	100 (92.4–100)	100 (96.0–100)	100 (34.2–100)
Cirrhosis	99.2 (97.0–99.8)	99.0 (94.7–99.8)	96.3 (81.7–99.3)	100 (94.6–100)	100 (70.1–100)	98.9 (94.2–99.8)	100 (91.0–100)	98.0 (89.5–99.6)	100 (56.6–100)
Decompensated cirrhosis	96.0 (80.5–99.3)	92.3 (66.7–98.6)	100 (70.1–100)	75.0 (30.1–95.4)	100 (34.2–100)	100 (43.9–100)	100 (34.2–100)	100 (20.7–100)	NA
Treatment-naive	98.2 (95.6–99.3)	96.5 (90.2–98.8)	93.8 (79.9–98.3)	97.8 (88.7–99.6)	100 (67.6–100)	99.1 (95.2–99.8)	100 (92.4–100)	98.4 (91.3–99.7)	100 (64.6–100)
Treatment-experienced	100 (98.5–100)	100 (96.1–100)	100 (83.9–100)	100 (94.9–100)	100 (43.9–100)	100 (96.9–100)	100 (91.0–100)	100 (95.5–100)	NA

Among genotype 3–infected patients, the best SVR was achieved in those treated with DAC + SOF (SVR, 97.4%; 95% CI, 94.7–98.7%), followed by SOF/VEL (SVR, 92.9%; 95% CI, 77.4–98.0%) and LDV/SOF (SVR, 87.5%; 95% CI, 52.9–97.8%). However, there was no significant difference in SVR12 between all three DAA regimens (Table 3). Among cirrhotic, treatment-experienced, genotype 3–infected patients who received 12 weeks of therapy, SVR12 was significantly higher in patients who were co-administered with RBV (98.1%, 52 of 53) than those did not (76.9%, 10 of 13) (*p* = 0.02). SVR12 was achieved in all treatment-experienced patients with cirrhosis who received extending treatment duration to 16 or 24 weeks. In patients with available subgenotype data (*n* = 244), no significant difference in treatment response was observed between patients with genotype 3a (97.2%, 210 of 216) and genotype 3b (89.3%, 25 of 28) patients. However, the SVR12 rate was significantly higher in treatment-experienced patients with genotype 3a infection compared with those with genotype 3b (99.1% vs. 57.1%; *p* = 0.001) (Fig. 3b). Likewise, cirrhotic patients with genotype 3a had slightly higher SVR12 than those with genotype 3b (95.7% vs. 88.2%).

**Table 3:** Sustained Virologic Response Rates (95% Confidence Intervals) in Patients infected with HCV Genotype 3

	Genotype 3 SVR, % (95% CI)	Genotype 3a SVR, % (95% CI)				Genotype 3b SVR, % (95% CI)			
	All patients	All patients	LDV/SOF	DAC + SOF	SOF/VEL	All patients	LDV/SOF	DAC + SOF	SOF/VEL
Patients who complete, N	303	216	7	186	23	28	NA	27	1
All durations	96.7 (94.0–98.2)	97.2 (94.1–98.7)	85.7 (48.7–97.4)	98.4 (95.4–99.5)	91.3 (73.2–97.6)	89.3 (72.8–96.3)	NA	88.9 (71.9–96.1)	100 (20.7–100)
12-wk treatment	96.3 (92.8–98.1)	96.9 (92.9–98.6)	80.0 (37.6–96.4)	98.5 (94.6–99.6)	91.3 (73.2–97.6)	88.9 (67.2–96.9)	NA	88.2 (65.7–96.7)	100 (20.7–100)
16-wk treatment	93.5 (79.3–98.2)	93.8 (71.7–98.9)	NA	93.8 (71.7–98.9)	NA	83.3 (43.6–97.0)	NA	83.3 (43.6–97.0)	NA
24-wk treatment	100 (93.8–100)	100 (91.4–100)	100 (34.2–100)	100 (91.0–100)	NA	100 (51.0–100)	NA	100 (51.9–100)	NA
SVR in the following subgroups									
No cirrhosis	99.0 (94.8–99.8)	100 (95.1–100)	100 (34.2–100)	100 (94.6–100)	100 (61.0–100)	90.9 (62.3–98.4)	NA	90.9 (62.3–98.4)	NA
Cirrhosis	95.5 (91.6–97.6)	95.7 (91.0–98.0)	80.0 (37.6–96.4)	97.5 (92.8–99.1)	88.2 (65.7–96.7)	88.2 (65.7–96.7)	NA	87.5 (64.0–96.5)	100 (20.7–100)
Decompensated cirrhosis	88.4 (75.5–94.9)	86.7 (70.3–94.7)	100 (20.7–100)	91.7 (74.2–97.7)	80.0 (37.6–96.4)	100 (61.0–100)	NA	100 (61.0–100)	NA
Treatment-naive	96.8 (92.7–98.6)	95.5 (89.8–98.0)	66.7 (20.8–93.9)	96.6 (90.6–98.8)	94.4 (74.2–99.0)	100 (84.5–100)	NA	100 (84.5–100)	NA
Treatment-experienced	96.6 (92.3–98.5)	99.1 (94.8–99.8)	100 (51.0–100)	100 (96.2–100)	80.0 (37.6–96.4)	57.1 (25.0–84.2)	NA	50.0 (18.8–81.2)	100 (20.7–100)

Among genotype 2–infected patients, all 14 patients in the mITT analysis, including three patients with cirrhosis and two treatment-experienced patients achieved SVR12 with 12− or 24 − week of DAC + SOF ± RBV and SOF/VEL monotherapy (Table 4). In genotype 4–infected patients, SVR12 was achieved by 90% of the patients who received DCV + SOF regimens, and another patient achieved SVR12 with SOF/VEL monotherapy for 12 weeks (Table 4).

**Table 4:** Sustained Virologic Response Rates (95% Confidence Intervals) in Patients infected with HCV Genotype 2, 4, and 6

	Genotype 2 SVR, % (95% CI)			Genotype 4 SVR, % (95% CI)			Genotype 6 SVR, % (95% CI)			
	All patients	DAC + SOF	SOF/VEL	All patients	LDV/SOF	DAC + SOF	All patients	LDV/SOF	DAC + SOF	SOF/VEL
Patients who complete, N	14	10	4	11	1	10	152	36	109	7
All durations	100 (78.5–100)	100 (72.2–100)	100 (51.0–100)	90.9 (62.3–98.4)	100 (20.7–100)	90.0 (59.6–98.2)	96.7 (92.5–98.6)	97.2 (85.8–99.5)	96.3 (90.9–98.6)	100 (64.6–100)
12-wk treatment	100 (77.2–100)	100 (70.1–100)	100 (51.0–100)	87.5 (52.9–97.8)	100 (20.7–100)	85.7 (48.7–97.4)	97.1 (92.9–98.9)	97.0 (84.7–99.5)	97.0 (91.5–99.0)	100 (64.6–100)
16-wk treatment	NA	NA	NA	NA	NA	NA	100 (34.2–100)	NA	100 (34.2–100)	NA
24-wk treatment	100 (20.7–100)	100 (20.7–100)	NA	100 (43.9–100)	NA	100 (43.9–100)	90.0 (59.6–98.2)	100 (43.9–100)	85.7 (48.7–97.4)	NA
SVR in the following subgroups										
No cirrhosis	100 (74.1–100)	100 (64.6–100)	100 (51.0–100)	100 (61.0–100)	100 (20.7–100)	100 (56.6–100)	96.8 (90.9–98.9)	100 (82.4–100)	95.8 (88.5–98.6)	100 (43.9–100)
Cirrhosis	100 (43.9–100)	100 (43.9–100)	NA	80.0 (37.6–96.4)	NA	80.0 (37.6–96.4)	96.6 (88.5–99.1)	94.4 (74.2–99.0)	97.3 (86.2–99.5)	100 (51.0–100)
Decompensated cirrhosis	100 (20.7–100)	100 (20.7–100)	NA	100 (20.7–100)	NA	100 (20.7–100)	100 (67.6–100)	100 (34.2–100)	100 (56.6–100)	100 (20.7–100)
Treatment-naive	100 (75.8–100)	100 (67.6–100)	100 (51.0–100)	83.3 (43.6–97.0)	NA	83.3 (43.6–97.0)	99.1 (94.8–99.8)	100 (88.6–100)	98.6 (92.3–99.7)	100 (61.0–100)
Treatment-experienced	100 (34.2–100)	100 (34.2–100)	NA	100 (56.6–100)	100 (20.7–100)	100 (51.0–100)	91.3 (79.7–96.6)	83.3 (43.6–97.0)	92.3 (79.7–97.3)	100 (20.7–100)

Among genotype 6–infected patients, all treatment regimens studied had similarly high SVR12 rates, ranging from 96.3 to 100%, with no significant difference between DAC + SOF, LDV/SOF, and SOF/VEL regimens (Table 4). Comparable SVR12 rates were observed in patients with (96.6%, 57 of 59) and without (96.8, 90 of 93) cirrhosis. The overall SVR rate for treatment-experienced patients with cirrhosis was 91.3% (21 of 23), and rates were comparable between patients who were co-administered with RBV (85.7%, 6 of 7) and those did not (93.8%, 15 of 16). Among patients with cirrhosis and prior HCV treatment, SVR12 rates did not differ between patients receiving 12 weeks of therapy (88.2%, 15 of 17) and those extending treatment duration to 16 or 24 weeks (100%, 6 of 6).

### Treatment failure

Twenty patients did not achieve SVR12. Two patients failed to achieve SVR12 due to early treatment discontinuation without evidence of virological failure. Two additional patients relapsed at post-treatment week 24 and 52 after achieving SVR12. Treatment failure occurred with similar frequency among patients treated with DCV + SOF ± RBV (2.1%), LDV/SOF ± RBV (2.6%), and SOF/VEL alone (3.5%).

We examined baseline variables that might predict treatment failure. Univariate logistic regression analysis indicated significantly higher risks of treatment failure among patients with more advanced liver disease, as indicated by baseline MELD score ≥ 16 (odds ratio [OR], 6.58; 95% CI, 1.37–31.6), CTP class B or C (OR, 5.24; 95% CI, 1.84–14.9), albumin levels < 3.5 g/dL (OR, 3.96; 95% CI, 1.62–9.67), total bilirubin ≥1.2 mg/dL (OR, 4.27; 95% CI, 1.68–10.8), or platelet counts < 100 × 10^9^/L (OR, 3.19; 95% CI, 1.30–7.82). Only baseline bilirubin ≥1.2 mg/dL was significantly associated with increased risk of treatment failure on multivariate analysis.

### Safety and tolerability

The most common adverse events were non-specific, such as fatigue (11%), insomnia (2.7%), headache (2.5%), gastrointestinal events (1.8%), rash (1%) and arthralgia (0.7%). Liver-related grade 3–4 laboratory abnormalities were uncommon; ALT elevations were reported in 3 patients (0.1%) and elevated total bilirubin in 41 (4%). Among the cirrhosis population, 26.6% of patients receiving RBV experienced any clinical adverse events, compared with 13.1% of those without the use of RBV (*p* = 0.002). However, most adverse events in the two groups were mild in severity. All patients with stage 3–4 chronic kidney disease, patients coinfected with either HBV or HIV, and liver transplant recipients tolerated the DAAs well, with none of the patients reporting any serious adverse events.

Of the patients who initiated therapy, 874 (86%) completed 12 weeks of treatment, 41 (4%) completed 16 weeks, and 98 (9.6%) completed 24 weeks. Four patients stopped treatment after 14–20 weeks per physician choice, and four patients discontinued treatment before week 4 due to non-hepatic adverse events (1 required hospitalization for sepsis, 1 underwent emergency surgery for a perforated peptic ulcer, 1 developed an allergic skin reaction, and 1 experienced emotionally unstable personality disorder). Fifty-three patients (51 DCV + SOF and 2 LDV/SOF) were excluded from the mITT population, of whom 52 patients were lost to follow-up, and one patient died after the completion of 12-week therapy (Fig. 1). Fifty-three excluded patients received therapy for ≥12 weeks and had only baseline HCV RNA data available.

## Discussion

This multicenter Thai cohort provided clinically relevant information on the effectiveness and safety of SOF plus the NS5A inhibitors, e.g., DAC, LDV, or VEL in a large cohort of patients infected with diverse HCV genotypes and high proportions of treatment-experienced patients and those with cirrhosis, including decompensated cirrhosis.

The introduction of the nucleotide analogue NS5B polymerase inhibitor SOF marked the important milestone in HCV therapy [4]. Subsequently, several other HCV NS5A inhibitors were developed, and a combination of them with SOF have shown improvements in antiviral efficacy with high resistance barriers and good safety profiles. In phase III studies, the once-daily oral combination of SOF with NS5A inhibitors, e.g., DAC, LDV or VEL reported SVR12 rates > 95% of HCV patients across different populations [5–13]. However, clinical trials typically include highly selected patient populations with fewer comorbidities and high adherence rates. Hence, there remains a need to determine whether these high rates of SVR would be achieved in real-world settings. Our results of an unselected patient population treated in a variety of treatment settings exhibit high overall effectiveness of SOF with NS5A inhibitors in real-life practice. Among genotype 1–infected patients, there was no significant difference in SVR12 rates across all SOF containing regimens, and SVR12 rates > 95% were achieved even in subgroups such as treatment-experienced or cirrhotic patients. Overall, our subgroups of patients infected with genotype 1a and 1b achieved similarly high rates of SVR12. Treatment with SOF plus LDV, DAC, or VEL, were recommended for 12 weeks by national treatment guideline during the study period. Nevertheless, extending treatment duration from 12 weeks to 16 or 24 weeks was considered to improve the SVR rate in some difficult-to-treat populations, such as patients with cirrhosis and prior treatment experience. Excellent SVR rates were observed with extending treatment duration; however, it was not significantly different from those treated with 12-week regimens. Similarly, adding RBV to 12 weeks of therapy did not improve SVR in this subgroup. The data confirm the high effectiveness of SOF and the NS5A inhibitors for 12 weeks in genotype 1 − infected patients, as reported in other real-world studies [14–18, 25].

Our cohort included 314 patients with genotype 3 infection, who achieved surprisingly high overall SVR rates of 96.7%. This high SVR12 rate was driven by genotype 3a − infected patients who had much higher SVR12 (97.2%) than genotype 3b − infected patients (89.3%), particularly with prior treatment experience (57.1%) or presence of cirrhosis (88.2%). This finding was consistent with a recent clinical trial in Asian patients treated with SOF/VEL, which showed that the SVR12 rate among patients with genotype 3b, particularly those with cirrhosis, was lower than that observed in genotype 3a − infected patients [31]. This was due to the higher prevalence of NS5A resistance-associated substitutions at baseline in those with HCV genotype 3b infection [31]. However, resistance testing is not routinely performed in our practice, and therefore, data on the presence of resistance mutations at relapse to any of the DAAs used are not available. Notably, LDV/SOF and RBV were also used in our practice for the limited number of patients infected with genotype 3a, which achieved an excellent SVR rate (100%), with the 24-week regimen. Overall, these results support the combined use of SOF with DAC, LDV, or VEL in patients with genotype 3 infection [21–23], but suggest that genotype 3b − infected patients with cirrhosis or prior HCV therapy had lower SVR rates with the pan-genotypic DAC + SOF or SOF/VEL regimens.

Previously, no optimal treatment was available for genotype 2–infected patients with the first-generation DAAs. However, physicians currently have excellent options in these patients: the two recommended combination regimens are the fixed-dose of DAC + SOF or SOF/VEL. Our observational cohort study of 16 patients who received DAC + SOF ± RBV for 12 or 24 weeks, or SOF/VEL monotherapy for 12 weeks, reported SVR rates of 100% for both regimens. Consistent with previous clinical trials [8, 11, 12], existing real-world data support that both combination regimens should replace SOF plus RBV as the standard of care in genotype 2 infection [4, 22]. Among 13 patients with genotype 4 infection, they had the lowest SVR rates in our study, just as in a real-world study [32]. Our data revealed that DAC + SOF and RBV for 12 weeks had a lower SVR rate (87.5%) than DAC + SOF for 24 weeks (100%). However, it should be noted that this patient population was small. Recently, a large real-world experience of DAC + SOF for 12 weeks showed high SVR rates in genotype 4-infected patients with different stages of liver disease [20].

HCV genotype 6 is mostly geographically restricted to Southeast Asia, making it a less-evaluated genotype in the clinical trials done primarily in Western Countries. Thus, the results are not readily applicable to large populations with this genotype in endemic areas. In our cohort of 166 patients with genotype 6 infection, treatment with DAC + SOF, LDV/SOF, and SOF/VEL resulted in remarkably high SVR rates, approaching the rates reported in clinical trials and other real-world experiences [11, 25, 26, 33]. DAC + SOF and LDV/SOF yielded above 95% SVR12 rates for the treatment-naïve group and the non-cirrhotic group, while SOF/VEL provided 100% SVR12 rates regardless of cirrhotic status and prior treatment experience. Although SVR rates for SOF/VEL may have been higher than those of DAC + SOF and LDV/SOF, our analysis showed no significant difference, probably due to the small sample size of patient subgroups. In genotype 6-infected patients with cirrhosis and prior HCV therapy, we also evaluate the utility of adding RBV and extending treatment on SVR rates in this population. Our results showed that adding RBV and extending treatment duration to 16 or 24 weeks did not improve SVR12 in this subgroup. Our finding is encouraging that SOF plus NS5A inhibitors can be effective options for patients with genotype 6 infection, though further study with a larger sample size is warranted.

In this real-world cohort, cirrhotic and non-cirrhotic patients achieved similarly high rates of SVR12. Our analysis further demonstrated that SOF, combined with the NS5A inhibitors, was well-tolerated and highly effective in patients with cirrhosis. Overall SVR rates were achieved by 97.6% with DAC + SOF, 96.9% with LDV/SOF, and 94.7% with SOF/VEL in cirrhotic patients. In the current era of DAAs, genotype 3 − infected patients with cirrhosis and prior HCV therapy have emerged as a more difficult-to-cure population where less evidence-based guidance may be available. Real-world physicians may make empiric decisions to extend treatment, add RBV, or both. In our analysis for genotype 3, adding RBV to the 12-week regimen improved SVR rates in patients with cirrhosis and prior HCV therapy. Furthermore, extending treatment duration to 16 or 24 weeks has greatly improved SVR12 rates in this patient population. These response rates were higher than those observed in patients with genotype 3 HCV infection and cirrhosis treated with DAC + SOF for 12 weeks in ALLY− 3 (SVR12 rate 63%) [9], and with the addition of RBV or an extended duration of DAC + SOF in ALLY− 3+ (SVR rates 83% or 89%, respectively) [10]. This difference could be explained by the fact that our treatment regimens were not randomly assigned, and it might be caused by physicians’ common practice of adding RBV to regimens or extending treatment duration for harder-to-treat patients.

The SOF combined with DCV, LDV, or VEL ± RBV regimens has shown to be safe and effective in clinical trials when used in patients with decompensated cirrhosis [13, 34, 35]. However, real-life data on HCV treatment with DAA in decompensated cirrhosis patients are conflicting. Results from an international multicenter cohort study reported similar SVR12 rates among patients with compensated and decompensated cirrhosis [36]. In contrast, a large Spanish real-life cohort showed significantly lower rates of SVR12 in patients with CTP class B/C compared with those with CTP class A cirrhosis [37]. In our cohort of 521 patients with cirrhosis, the SVR12 rate was lower in patients with CTP class B/C compared to those with CTP class A. Correspondingly, indicators of more advanced disease, such as low platelet count and low albumin level were significantly associated with increased risk of treatment failure, advocating for earlier treatment of such patients, before the onset of advanced liver disease.

Our analysis further demonstrated that patients with decompensated cirrhosis experienced an improvement in liver function with the treatment-induced HCV clearance. This observation supports earlier studies showing the clinical benefit of attaining SVR leading to avoid liver transplant in patients with decompensated cirrhosis [38, 39]. However, these studies provided evidence of some patients with decompensated cirrhosis ending up in ‘MELD purgatory’ after achieving viral clearance [38, 40]. Of these, many patients still require liver transplantation, but chances of organ allocation are reduced due to improved MELD score. Consistent with other studies in similar populations [37, 41], our finding confirmed that DAA treatment in decompensated individuals was less effective than in liver transplant recipients but still achieves SVR in a majority of cases. Based on this, it remains unclear if patients with decompensated cirrhosis benefit from immediate antiviral treatment, and which patients should possibly undergo transplant first. Recently, a simulation of the liver transplant model identifies the subset of patients with decompensation who are likely to derive benefit from treatment and who should be urgently considered for DAA therapy [42]. The results suggested that patients with decompensation and a MELD score of ≤23–27 should receive HCV therapy while awaiting liver transplantation [42]. Accordingly, treating HCV before transplantation was reported to be cost-effective for patients without HCC with a MELD score ≤ 20, while antiviral treatment after transplantation was cost-effective in those with a MELD score > 20 [43]. Nevertheless, clinicians should consider the potential risks and benefits of treating patients with advanced liver disease on a case-by-case basis through a multidisciplinary discussion.

While SOF is the cornerstone of most current anti-HCV regimens, its efficacy and safety in patients with chronic kidney disease are not fully established. This agent is eliminated by the kidneys and not approved for patients with eGFR < 30 mL/min/1.73 m^2^. However, the significant and growing need to treat HCV infection in patients with advanced chronic kidney disease has led some clinicians to administer SOF in patients with severe renal impairment. According to a recent meta-analysis of 11 clinical studies involving 264 patients with eGFR < 30 mL/min/1.73 m^2^, the pooled SVR12 rate was 89% (95% CI, 82–95%) with the SOF containing regimens [44]. Efficacy outcome was emphasized by our analysis of 84 patients with stage 3–4 chronic kidney disease, who achieved high SVR12 rates of 98.7%–100%. Of note, our study included only six patients with eGFR < 30 mL/min/1.73 m^2^, who were well tolerated with regimens containing a standard dose of SOF. Further well-design studies with a larger sample size would be beneficial to clinicians and patients.

The drug-drug interaction of antiviral agents becomes a vital consideration issue for HCV-infected individuals with comorbidities that require concomitant medications, such as HCV/HIV coinfection or immunosuppression after liver transplantation. No clinically significant interaction was observed in our subgroups, indicating that SOF combined with NS5A inhibitors were safe when administered with common antiretroviral agents or antirejection drugs. However, further information needed to be collected. Clinicians managing these special populations should actively assess co-medications before selecting a DAA combination to treat HCV infection.

This study had some limitations related to its real-world observational design. First, 5.2% of the studied patients lacking SVR data were excluded from the mITT analysis; however, the impact on treatment response rate was likely minimal given the small number and that such excluded patients differed minimally from patients with available SVR data. Second, the limited requirements for data capture in clinical practice may lead to underreporting of adverse events. Nonetheless, it is unlikely that clinically relevant adverse events were missed. Finally, given that this was the real-world setting, some patients received regimens outside of current guidelines.

## Conclusion

This large observational study has shown that SOF combined with NS5A inhibitors are highly effective with excellent safety profiles in the diverse patient population managed in routine practice. SVR rates parallel those achieved into clinical trials, demonstrating how effectively these regimens have translated into clinical practice. This real-world experience in Thailand supports current evidence-based guidelines for the use of SOF combined with DAC, LED, or VEL for 12 weeks as effective treatment options for genotypes 1 and 6. In patients infected with genotype 3, SOF plus DCV or VEL for 12 weeks produced high SVR rates. Adding RBV and extending treatment duration of DAC + SOF potentially improved SVR rates in genotype 3-infected patients with cirrhosis and prior treatment experience. Our finding also supports that SOF combined with DAC or VEL for 12 weeks can be used as an optimal treatment for genotype 2-infected patients. The 12-week therapy with SOF plus DAC is potentially useful for patients with genotype 6. However, given a small number of genotype 2-and 4-infected patients within the subpopulation, more corporate data is necessary to draw a firm conclusion.

## Data Availability

The data used and/or analyzed during the study are available from the corresponding author on reasonable request.

## Abbreviations

CI: Confidence interval
CTP: Child-Turcotte-Pugh
DAAs: Direct-acting antiviral agents
DAC: Daclatasvir
eGFR: Estimated glomerular filtration rate
HBV: Hepatitis B virus
HCC: Hepatocellular carcinoma
HCV: Hepatitis C virus
HIV: Human immunodeficiency virus
LDV: Ledipasvir
MELD: The model of the end-stage liver disease
mITT: The modified intention-to-treat
NS5A: Nonstructural protein 5A
RBV: Ribavirin
SOF: Sofosbuvir
SVR: Sustained virologic response
VEL: Velpatasvir
